# Special Issue on Molecular Research Efforts in Urothelial Carcinoma: Summary of Included Topics

**DOI:** 10.3390/ijms20153790

**Published:** 2019-08-02

**Authors:** Georg C. Hutterer

**Affiliations:** Department of Urology, Medical University of Graz, Auenbruggerplatz 29, 8036 Graz, Austria

## Abstract

This Special Issue of *International Journal of Molecular Sciences* (IJMS) covers one of the most intriguing and emerging fields in terms of molecular oncology and uro-oncologic research efforts over the recent years, namely urothelial carcinoma of the bladder (UCB), as well as urothelial carcinoma of the upper urinary tract (UTUC). A total of 8 articles published in this Special Issue highlight the current progress in molecular oncology and cancer genetics in UCB, including a wide range of research topics, such as FGFR-inhibitors, sarcopenia in UCB, molecular predictors of response following neoadjuvant chemotherapy, exercise cardiac training impacts in the murine UCB model, Obatoclax, tropomyosins as potential biomarkers, immunotherapeutic approaches, as well as a transcriptional analysis of immunohistochemically defined UCB-subgroups. Find a brief summary of the respective articles below.

## 1. Overcoming Paclitaxel-Resistance in FGFR1-Overexpressing Urothelial Carcinoma via FGFR-Inhibition

Paclitaxel (PTX), a single-agent taxane used to treat advanced unresectable or metastatic UC after the failure of platinum-based chemotherapy, is not sufficient to inhibit tumor progression and drug resistance in the long term [1]. Epithelial-to-mesenchymal transition (EMT) induced by fibroblast growth factor receptor (FGFR)1 signaling has been proposed as a mechanism of PTX-resistance, whereby it is currently unclear whether or not it can be overcome by FGFR1-inhibition [2,3].

The present study investigated whether FGFR1-overexpression contributes to PTX-resistance and whether FGFR-inhibition is able to enhance PTX efficacy in UC [4]. The effects of PTX combined with the FGFR-inhibitor BGJ398 were evaluated in UC cell lines by flow cytometry, Western blot analysis, cell viability, migration, colony forming assays, and RNA interference. The authors report that PTX + BGJ398 were able to induce cell cycle arrest and apoptosis in UC cells with mesenchymal characteristics, which was accompanied by the downregulation of cyclin D1 protein and upregulation of gamma-histone 2A family member X and cleaved poly(ADP-ribose) polymerase. Additionally, PTX + BGJ398 synergistically suppressed UC cell migration and colony formation via regulation of EMT, while FGFR1-knockdown enhanced the antitumor effect of PTX.

These findings might provide a basis for the development of effective strategies for overcoming PTX-resistance in advanced UC through inhibition of FGFR1-signaling [4]. The issue is particularly important, since patients with advanced or metastatic UC show, in general, a progression of the disease after receiving 6 to 8 months of platinum-based first-line chemotherapy. Furthermore, the response rates to second-line PD-1/L1 inhibitors are as low as 13–21%, with limited options after disease progression [5,6].

## 2. Metabolic and Molecular Basis of Sarcopenia in Urothelial Carcinoma

Sarcopenia, which is characterized by a degenerative and systemic loss of skeletal muscle mass, represents a multifactorial syndrome that (amongst others) can be found accompanying advanced or progressive cancers, as well as in patients with cancer cachexia [7]. A growing body of evidence has shown that sarcopenia is significantly associated with higher rates of treatment-related complications and worse prognosis in patients with advanced UC, as well as UTUC [8]. Moreover, recently published studies reported that a post-therapeutic increase in skeletal muscle mass predicts favorable prognosis in UC patients [9].

The authors provide a systematic and detailed overview of the metabolic and molecular basis of cancer cachexia and sarcopenia, describing the PI3K/Akt signaling pathway as a key regulator of skeletal muscle homeostasis [8]. Additionally, oxidative stress mechanisms are described as promotors of skeletal muscle depletion by increasing protein degradation and inducing apoptosis of myofibers by damaging mitochondrial DNA. Finally, possible mechanisms of increasing skeletal muscle mass during the course of cancer treatment, such as hypertrophic changes in myofibers or skeletal muscle regeneration via the activation of satellite cells, are discussed.

The respective manuscript is of great clinical relevance, particularly since the prevalence of sarcopenia has recently been shown to be much higher than thought, ranging from 15% at 65 years to 50% at 80 years [8,9]. Moreover, this condition is burdensome for affected patients, being associated with poor physical performance, a high risk of fall and fracture, high rates of morbidity from infectious diseases, metabolic syndrome, insulin resistance, cardiovascular diseases, and high rates of mortality [10]. Since sarcopenia involves the general health, as well as the nutritional status of a patient, systemic inflammation, metabolic abnormalities, oxidative stress, and cancer progression, it might represent a potentially useful prognostic biomarker in the management of UC in the future.

## 3. Molecular Predictors of Complete Response Following Neoadjuvant Chemotherapy in Urothelial Carcinoma of the Bladder and Upper Urinary Tract

While neoadjuvant chemotherapy (NAC) followed by radical cystectomy (RC) represents an established standard of care in UCB, strong scientific evidence for a similar approach is lacking in UTUC. The longest survival is observed in patients with complete response (pT0) on pathological examination of the RC specimen, but the impact of delayed RC in nonresponders may be detrimental [11]. The rate of pT0 following NAC in UTUC is considerably lower than that in UCB, due to differences in access and instrumentation.

A variety of potential biomarkers have been evaluated to predict response to Cisplatin-based chemotherapy, including DNA repair genes (*ATM*, *RB1*, *FANCC*, *ERCC2*, *BRCA1*, *ERCC1*), regulators of apoptosis (survivin, Bcl-xL, emmprin), receptor tyrosine kinases (EGFR, erbB2), and genes involved in cellular efflux (*MDR1*, *CTR1*), in addition to molecular subtypes (basal, luminal, p53-like).

The current state of the literature on the prediction of response to NAC based on the presence of these biomarkers is discussed in great detail in this review [12]. As the authors conclude, incorporating biomarkers into recent clinical decision-making could potentially predict response to NAC and avoid chemotherapy in patients who are unlikely to respond, thus expediting surgery and decreasing the cost and toxicity associated with NAC. The covered topic is of particular importance, considering the extraordinary aggressiveness and bad prognosis of muscle-invasive UTUC, as evidenced by 5 year cancer-specific survival (CSS) rates <50% in pT2/T3 disease and <10% in pT4 disease [13].

Moreover, Lotan et al. developed a decision analysis model to evaluate the cost effectiveness of a biomarker-based approach to NAC using a DNA repair gene panel (*ATM*, *RB1*, *FANCC*), *ERCC2*, and RNA subtypes [14]. The respective model compared RC alone, unselected NAC followed by RC, and biomarker-directed NAC followed by RC. The authors report that the DNA repair gene panel-based approach was the most cost-effective strategy, with a mean overall survival (OS) of 3.14 years and expenses of $31,482/life year. Under this model, 38% of patients would go on to receive NAC. This approach increased the mean OS by 5.2 months and 1.6 months, compared to RC alone and compared to a scenario where all patients received NAC.

In conclusion, the use of biomarkers to stratify NAC administration based on predicted response might represent a cost-effective option, although a prospective validation is needed before this approach would be ready for daily clinical routine use.

## 4. Exercise Training Impacts Cardiac Mitochondrial Proteome Remodeling in Murine Urothelial Carcinoma

Since cardiac dysfunction secondary to cancer may exert a negative impact in patients’ tolerance towards therapeutics, quality of life, and survival [15], the aim of the recent study was to evaluate the potential therapeutic effect of exercise training on the heart in the setting of cancer after diagnosis [16]. The authors report the molecular pathways in heart mitochondria from a murine model of chemically-induced UC submitted to 8 weeks of high intensity treadmill exercise, characterized using mass spectrometry-based proteomics.

Protective effects of high intensity exercise training in preventing left ventricle diastolic dysfunction, fibrosis, and structural derangement observed in tumor-bearing mice are described. In more detail, a variety of cardioprotective changes promoted by exercise training (e.g., enhancement of cardiac contractile function, angiogenesis, changes in fibrillary collagen content and organization, enhanced neuronal nitric oxide synthase signaling within myocytes, and activation of the IGF-1/PI3K/Akt/mTOR pathway, as well as mitochondrial improvements) have been reported [17]. At the mitochondrial level, the authors were able to demonstrate that tumor-bearing mice presented lower functional mitochondria, as suggested by lower ATP synthase and citrate synthase activities and OXPHOS subunits levels. Exercise training counteracted the lower ability to produce ATP observed in the heart of animals with UC and induced the up-regulation of fatty acid oxidation and down-regulation of the biological process of cardiac morphogenesis.

Taken together, the recent data strongly support the prescription of exercise training after cancer diagnosis for a better management of disease-related cardiac dysfunction [16]. Notably, an efficient energetic substrate handling, improved muscle contraction, and down-regulation of cardiac morphogenesis were among the most important molecular alterations promoted by high intensity chronic exercise training.

## 5. Enhancement of Cisplatin-Induced Apoptosis and Reduction of the Clonogenicity of Muscle Invasive Bladder Cancer Cells via Obatoclax, a BH3 Mimetic

Several studies have determined that expression levels of Bcl-2 and/or Bcl-xL, pro-survival molecules which are associated with chemoresistance, are elevated in patients with muscle invasive UCB [18,19]. The goal of the recent analysis was to clarify whether combining Obatoclax, a BH3 mimetic which inhibits pro-survival Bcl-2 family members, is able to improve responses to Cisplatin chemotherapy, a standard of care treatment for muscle invasive UCB [20].

The background of this study is an increasing development of drugs which target the pro-survival members of the Bcl-2 family to combat intrinsic resistance to first-line chemotherapy in various cancer types [21,22]. Different approaches for the inhibition of the pro-survival members of the Bcl-2 family have been reported, including the development of anti-sense drugs and synthetic peptides [23,24,25]. However, BH3 mimetics currently appear to be among the most promising ones [21].

Three muscle invasive UCB cell lines (T24, TCCSuP, 5637) were treated with Obatoclax alone or in combination with Cisplatin and/or pre-miR-34a, a molecule which the authors have previously shown to inhibit UCB cell proliferation via decreasing Cdk6 expression. Proliferation, clonogenic, and apoptosis assays confirmed that Obatoclax is able to decrease cell proliferation and promote apoptosis in a dose-dependent fashion, whereby combination treatment experiments identified Obatoclax + Cisplatin as the most effective treatment modality. Moreover, Steele and colleagues were able to demonstrate that, in addition to being able to inhibit Bcl-2 and Bcl-xL, Obatoclax can also decrease cyclin D1 and Cdk4/6 expression levels, a finding that has not been previously reported [20].

In conclusion, Obatoclax was found to inhibit cell proliferation, promote apoptosis, and significantly enhance Cisplatin effectiveness in muscle invasive UCB cells via mechanisms that likely involve the inhibition of both pro-survival molecules, as well as cell cycle regulators. The recent analysis is of great importance, since the phenomenon of chemoresistance and thereby the non-response or significantly diminished response of patients towards chemotherapy represents a major problem in cancer treatment.

## 6. Tropomyosins as Potential Biomarkers for Urothelial Carcinoma of the Bladder

The development of accurate urinary biomarkers for the non-invasive detection of UCB could transform patient pathways by reducing the reliance on cystoscopy, which is equally burdensome for patients, as well as expensive for healthcare providers worldwide [26]. Thus, the design and composition of non-invasive diagnostic tools in UCB is of paramount importance and has become a global research goal [27]. Up to date, 6 biomarkers have been approved by the FDA for the detection and surveillance of UCB (NMP22, NMP22 BladderChek Test, BTA TRAK, BTA Stat, UroVysion® FISH, and ImmunoCyt). However, none of these biomarkers have been accepted into daily routine clinical practice so far, due to limited performance and/or evidence [28,29].

In this systematic and in-depth review article, the authors summarize and discuss potential biomarker candidates for UCB, namely the tropomyosin family of genes that are encoded by only 4 loci in the human genome, whereby the expression of these genes is tissue-specific [30]. Tropomyosins are responsible for manifold cellular functions, based upon their interplay with actin to maintain cellular processes, cellular integrity, and structure. The tropomyosin genes thus influence cellular motility, cell–cell adhesion, and extracellular interactions, as evidenced by their contribution to cancer cell metastasis via cell proliferation, migration, and invasion [31]. Moreover, Tropomyosins exhibit a large variety of splice forms and altered isoform expression levels have been associated with various types of cancer, including UCB. The interactions of tropomyosin isoforms with their actin-binding proteins influence the functional fate of individual cells, maintained by signaling pathways responsible for apoptosis, and hence the regulation of cell cycle and prevention of tumor growth in vivo. Oncogene-mediated alteration within signaling pathways affects Tropomyosin expression levels and mutagenic alterations mediated by Smad and MAPK pathways were found to play a pivotal role in the development of carcinogenesis in UCB [32]. As the authors state, the investigation of tropomyosin isoforms (currently >40) could mediate further advances in UCB diagnosis and risk stratification [30]. The question whether tropomyosin isoforms could be quantified as protein molecules within urine or genetic alterations within alternative splicing events of Tropomyosin mRNA, remains yet to be uncovered.

## 7. Aristolochic Acid and Immunotherapy for Urothelial Carcinoma

The genetic and epigenetic differences of UCB and UTUC, along with different responses to immunotherapy, were recently identified, indicating that the management of these diseases with similar principles and therapeutic approaches might be less effective [33]. Although both UCB and UTUC share tobacco exposure as one of their main risk factors, the consumption of aristolochic acid (AA), which had been a common component of Chinese medicines and certain weight-losing medications, was identified as a depictive environmental factor associated more with UTUC development, rather than UCB [34].

Aristolochic acid represents a natural alkaloid compound produced by the *Aristolochia* species of plants and is believed to cause its carcinogenicity through forming DNA adducts of deoxyadenosine-aristolactam, as well as A:T → T:A transversions in the *TP53* tumor suppressor gene [35,36]. Recent findings suggested that cancers with higher somatic mutations are associated with better treatment responses upon immune checkpoint blockade [37], therefore UTUC and AA-related biomarkers might serve as candidates, as well as potential prognostic predictors, for immunotherapy.

This informative review covers the current state of the literature on the clinical response of UCB and UTUC patients receiving immunotherapy and pinpoints various directions for refinement regarding better patient selection [38]. The topic discussed herein is of special clinical interest, since blocking inhibitory checkpoint molecules such as PD1 and its ligands, PD-L1 and PD-L2, are able to improve/restore the suppressed anticancer immune response [39]. Over the recent years this therapeutic strategy has led to the development of numerous inhibitory antibodies targeting immune checkpoints, known as immune checkpoint inhibitors, which, starting from the first PD-1 inhibitor, pembrolizumab (FDA approved in 2014), have been successfully implemented in therapies against various cancer types, including advanced/metastatic UCB [40]. However, there has not been direct evidence from any immune checkpoint inhibitor trial focusing on UTUC as disease entity or implementing AA-related biomarkers, so far.

## 8. Transcriptional Analysis of Immunohistochemically Defined Subgroups of Non-Muscle-Invasive Papillary High-Grade Upper Tract Urothelial Carcinoma

Recent molecular analyses based on gene expression profiles were able to demonstrate that both muscle-invasive, as well as non-muscle-invasive UCB, can be divided into different intrinsic molecular subtypes [41]. Although the classifications of muscle invasive UCB were named differently by respective research groups, they showed a significant overlap, common luminal and basal molecular subtypes were reported [42]. These distinct molecular subtypes showed differences in terms of prognosis, responsiveness to neoadjuvant chemotherapy, and targetable mutations. At the same time, gene expression profiles of non-muscle-invasive UCB showed 3 different clusters (class 1, 2, and 3), which differed in biologic signatures and in prognosis [43]. A recently performed comparison of microarray data, including 12 cases of UTUC and 20 cases of UCB, demonstrated that both tumors were unidentifiable at the gene expression level, except for pT3 tumors. It was also reported that UTUC was more enriched with luminal type genes compared to UCB [44]. However, the genomic landscape of UTUC has not been fully elucidated yet, mostly due to the relative rarity of the disease [45].

All the more, the respective research article warrants interest since the aim of the authors was to characterize the gene expression profiles of distinct subgroups of non-muscle-invasive papillary high-grade UTUC, classified by CK5/6 and CK20 expression levels (group 1 (CK5/6-high/CK20-low), group 2 (CK5/6-high/CK20-high), and group 3 (CK5/6-low/CK20-high)) [46]. The authors were able to demonstrate that expression of group 3 was predictive of worse prognosis of non-muscle-invasive papillary high-grade UTUC, whereby transcriptional analysis revealed >300 differentially expressed genes across the subgroups. Additionally, functional analyses of the genes identified cell adhesion and late cell cycle/proliferation signatures as a common process differentially enriched in group 3 compared to the other groups, which could explain its high-risk phenotype and might be used as a potential prognostic biomarker complementary to CK5/6 and CK20 in the future.
